# Latent trait or sum score: addressing measurement challenges in the prediction of self-rated symptom outcomes in psychological treatment

**DOI:** 10.3389/fpsyg.2026.1654996

**Published:** 2026-02-26

**Authors:** Nils Hentati Isacsson, Magnus Johansson, Viktor Kaldo

**Affiliations:** 1 Department of Clinical Neuroscience, Stockholm Health Care Services, Centre for Psychiatry Research, Karolinska Institutet, Stockholm, Sweden; 2 Division Built Environment, RISE Research Institutes of Sweden, System Transition, Gothenburg, Sweden; 3 Department of Psychology, Faculty of Health and Life Sciences, Linnaeus University, Växjö, Sweden

**Keywords:** digital mental health, ICBT, latent trait, machine learning, prediction, Rasch Measurement, treatment outcome

## Abstract

**Objective:**

Reliable and accurate measurement is fundamental to scientific progress; however, the dominant measurement practices in psychology, clinical psychology, and prediction research often lack rigor. Improving measures using Rasch Measurement Theory (RMT) offers advantages by fulfilling the key psychometric properties of unidimensionality, local independence of items, ordering of response categories, and invariance. Ordinal-level sum scores can be transformed into interval-level latent trait scores, thereby improving the measurement precision. However, the impact of using psychometrically advanced questionnaires with latent trait scores, as opposed to traditional sum scores, in predictive models is still unclear. This study evaluates whether using latent trait scores as predictors and outcomes, in accordance with RMT, improves predictive performance compared to using traditional sum scores when predicting treatment outcomes during psychological treatment.

**Methods:**

Self-rated symptom data from three different questionnaires, collected over the first 4 weeks of psychological treatment from 6,464 patients undergoing a 12-week treatment program, were used to predict post-treatment outcomes on the same questionnaires. This was done in two ways: (1) using sum scores as the questionnaires were originally developed and (2) using a reformulated, more psychometrically robust version of the questionnaires based on Rasch analysis, which was also shorter. The prediction models used were linear regression, Bayesian ridge regression, and random forest. Multiple imputations were used to address missing data, and nested cross-validation was employed for hyperparameter tuning and scoring.

**Results:**

Latent scores calculated using the psychometrically optimized shorter version, which comprises 23% of the full scale, showed similar predictive performance compared to the sum score of the full scale. Overall, there was a statistically significant but practically negligible difference of 0.007–0.008 in the root mean squared error (RMSE) when comparing the original sum score to the latent trait scores.

**Conclusion:**

Initial findings comparing psychometrically improved questionnaires with the original ordinal sum scores within a predictive framework indicate that using latent trait scores derived from these improvements showed the predictive performance similar to the sum score of the full scale. The small differences suggest that the improved versions remain valuable owing to their enhanced psychometric qualities and the reduction in response burden by using considerably fewer items. Further research is needed to explore the use of latent trait scores compared to ordinal sum scores in predictive research.

## Introduction

Reliable and accurate measurement is the cornerstone of scientific progress. The ability to define and, with validity, quantify phenomena consistently underpins the development of theories, the testing of hypotheses (Michell, 1997), and the application of findings to real-world challenges (Pendrill, 2018). Although not widely discussed in the field of psychological research, there has been a long-standing critique of the dominant measurement practices (Michell, 1997) with increased attention in recent years (Elson et al., 2023; Flake and Fried, 2020; Johansson et al., 2023).

Current practices often ignore issues regarding measurement (Flake et al., 2022), as exemplified by Lilienfeld and Strother (2020), seldom motivate the validity of instruments, and often rely on psychometric evaluations based on small samples (Elson et al., 2023). Furthermore, psychometric evaluations often rely on sum scores (McNeish and Wolf, 2020b) using Cronbach’s alpha (McNeish, 2018) to assess scale properties, an approach that has faced substantial methodological critique (McNeish, 2022). There is no widespread consensus on how to assess the psychometric quality; however, four key psychometric properties have been suggested as a minimal framework for guiding psychometric analyses: unidimensionality, local independence of items, ordering of response categories, and invariance (Christensen et al., 2013; Johansson et al., 2023; Kreiner, 2007).

**Figure 1:**
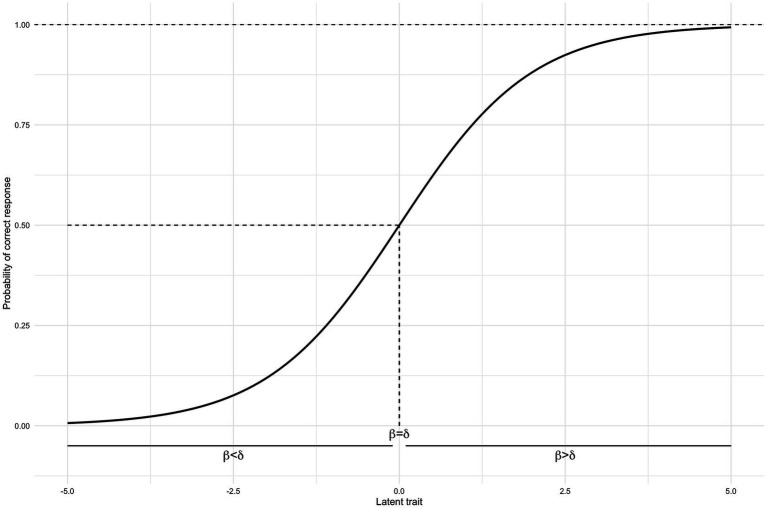
Probability of a correct response to a dichotomous item as a function of persons of varying proficiency. The latent trait β represents the latent ability of a person and varies between −5 and +5 on the logit scale in this figure. δ is the item difficulty, set to 0 in this figure.

Although these properties can be evaluated through various methods, Rasch Measurement Theory (RMT) offers the distinct advantage of treating the ordinal sum score as a sufficient statistic for measurement (Andrich and Marais, 2019). Rasch analysis models the probability of a response to an item based on a person’s *ability* or *trait (β)* and an item’s *difficulty* ( δ ), and the formulation of the model suggests that these two parameters can be separated, known as “specific objectivity.” Figure 1 shows the modeled probability of a correct response to a dichotomous item (Equation 1) with difficulty δ=0, depending on the varying level of a person’s latent trait β :


$$P(\text{Correct response}\;|\beta ,\delta )=\frac{e^{\beta -\delta }}{1+e^{\beta -\delta }}$$ (1)

Equation 1 is simplified in terms of notation and subscripts, as shown by Andrich and Marais (2019). Figure 1 illustrates that as a person’s trait increases, the probability of a correct response also increases. When the item difficulty (set to 0 in the figure) equals a person’s latent score β=δ=0 , the probability of a correct answer is 50% (see dashed lines in Figure 1).

As a person’s latent trait increases further from the item difficulty, β>δ , the probability of a correct answer increases toward 100%, and vice versa. Latent traits and item difficulties are both expressed in logit units (log-odds) with an arbitrary center or reference point (such as 0). In clinical psychology, a typical situation exemplified by Figure 1 could be a dichotomous item that indicates the presence of suicidal ideation. This question could be answered as yes or no with a numerical representation of 1 or 0, respectively. Such an item could be included in a questionnaire to measure the trait of depression; as this inferred trait increases (a more depressed patient), the probability of endorsing “yes” to suicidal ideation also increases. In short, the difficulty or δ of such an item would indicate that those with more of the latent trait of depression would indicate the presence (answer yes) of suicidal ideation. Specific objectivity essentially means that differences between items’ difficulties ( δ ) can be assessed independently of the current sample of respondents providing answers. Similarly, differences between persons ( β ) can be assessed independently of the difficulties of the items. When a set of items fulfills the criteria previously listed, Rasch analysis allows the transformation of an ordinal raw sum score into an interval-level latent trait score (latent score) for each person, with specific measurement error at each level of the scale. This represents an individual’s latent trait regarding what is being measured. In this article, a trait or latent score is the inferred *amount* of what is being measured, not an inherent trait.

In prediction research using self-rated symptom measures, as in psychological research, limited attention has been paid to measurement practices. Clinical prediction models for psychological treatment have the potential to improve treatment outcomes (Barkham et al., 2023; Forsell et al., 2019; Hentati Isacsson et al., 2024a). Therefore, improving these measures could have a significant impact on patients. There are several experiments investigating the impact of measurement error in prediction research involving self-rated symptoms. Jacobucci and Grimm (2020) showed using simulations that a predictor’s reliability can heavily influence the prediction performance (Jacobucci and Grimm, 2020), and medical prediction models have been shown to be less valid as a function of increased measurement error in predictors (Luijken et al., 2019). McNamara et al. (2022) took this one step further and simulated both measurement error in predictors and varying degrees of outcome misspecification (McNamara et al., 2022). The result was that the underlying non-linear relationship was not identified by non-linear models (e.g., random forest) or regression models, and non-linear models had indistinguishable performance. Furthermore, an attempt to attenuate measurement problems by increasing the sample size from 4,000 to 100,000 yielded negligible improvements in predictive performance. Thus, when the measurement error is high, the predictive performance plateaus at a low level compared to models using more reliable variables (McNamara et al., 2022), indicating heavy influence of reliability for both predictors and outcomes. This does not consider the validity of measurement. As such, it is perhaps surprising that the recently updated TRIPOD+AI statement for research on clinical prediction models does not mention measurement practices in relation to predictors or outcomes, except that it should be noted “…how and when they (predictors/outcomes) were measured…” (Collins et al., 2024). This is especially true in clinical psychology, where measurement error is an issue. Although using psychometrically sound measures is important and measurement reliability significantly affects predictions, there is limited empirical evidence that psychometrically refined measures offer the predictive advantages over summed ordinal scores based on a set of items that have not been subject to an adequate psychometric assessment. However, a few studies indicate that a measure validated using RMT can outperform a traditional measure in ROC/AUC classification performance, although these were for pregnancy tests and intensive care unit admissions (Fisher and Burton, 2010; Pendrill et al., 2023).

Recently, there has been an encompassing debate about the use of sum scores in psychological research (McNeish, 2024; McNeish and Wolf, 2020b; Sijtsma et al., 2024; Widaman and Revelle, 2023). In a simulation setting, for certain conditions, the sum score can correlate to a stronger degree with the true underlying latent score than the estimated latent score itself (Sijtsma et al., 2024). Furthermore, Sijtsma et al. (2024) showed using a simulation that the sum score can adequately represent the score from each item (similar to an estimated latent score). Thus, while the sum score can perform adequately both in a predictive context and for inference (Sijtsma et al., 2024), its current use of the sum score is seldom motivated properly (McNeish, 2024) in relation to psychometric properties such as dimensionality, validity, and invariance assessments. Therefore, the naive use of the sum score has a high risk for bias. However, when the sum score shows stochastic ordering, the latent score and sum can have similar performances in predicting an external variable (Sijtsma et al., 2024). Stochastic ordering means that, as the sum score increases, the latent variable also increases when conditioned on the sum score. While sum scores have pragmatic uses, a more psychometrically sound latent score is expected to improve predictive performance based on previous simulation studies. At the same time, empirical research on measuring change in psychometrically evaluated standardized tests of preschool children shows that the latent and ordinal sum scores show marginal differences (Bezruczko et al., 2016). Thus, the overall advantage of using latent scores over sum scores in an empirical predictive framework remains largely unexplored.

### Objectives

The aim of this study is to evaluate and compare predictive models using traditional sum scores and latent scores based on a reformulated, more psychometrically sound version of the questionnaires. Specifically, we investigated whether using the latent scores as predictors and outcomes with these reformulated questionnaires increases the predictive performance of the models predicting treatment outcomes in psychological treatment.

## Methods

This is a prospective prediction study using longitudinal observational data from a regular care clinic providing therapist-guided psychological treatment. Ethical approval was received from the regional ethical review board in Stockholm (Dnr: 2011/2091–31/3, amendment 2016/21–32, 2017/2320–32, and 2018/2550–32). The supplement contains the results data, code for analysis, and further details of the methods.

### Setting and participants

The participants (*n* = 6,464) were routine care patients at an Internet psychiatry clinic in Stockholm (Titov et al., 2018). They received 12 weeks of Internet-delivered Cognitive Behavioral Therapy (ICBT) for either major depressive disorder (*n* = 2,988), panic disorder (*n* = 1721), or social anxiety disorder (*n* = 1755). The treatments were guided by a licensed clinical psychologist and showed positive results (El Alaoui et al., 2015; Hedman et al., 2013, 2014). Each treatment consisted of condition-specific CBT techniques and weekly self-assessments of the primary symptoms. The data from all three treatments were pooled into a single dataset because this is beneficial for developing prediction models (Hentati Isacsson et al., 2024a; Zantvoort et al., 2024). The predicted outcome was the last self-assessment of the primary symptoms that occurred at treatment completion (post-treatment).

### Symptom data

The questionnaires used to assess the symptoms of each treatment were the Montgomery-Åsberg Depression Rating Scale-Self Report (MADRS-S) (Montgomery and Asberg, 1979) for major depressive disorder, the Panic Disorder Symptom Scale-Self Report (PDSS-SR) (Houck et al., 2002) for panic disorder, and the Leibowitz Social Anxiety Scale-Self Report version (LSAS-SR) (Fresco et al., 2001) for social anxiety disorder. These assessments were conducted at screening, before the start of treatment; on a weekly basis during treatment; and post-treatment. The post-treatment time point was the predicted outcome. Furthermore, a min-max transformation based on the questionnaires’ minimum and maximum scores was applied to each intervention sample individually to enable the aggregation of all three treatments (Cohen et al., 1999). The minimum ordinal sum score was 0 for all three questionnaires, and the maximum was 28, 54, and 144 for the PDSS-SR, MADRS-S, and LSAS-SR, respectively.

### Psychometric analyses

A prior analysis was conducted using a Rasch Measurement Theory (RMT) framework for all three questionnaires (MADRS-S, PDSS-SR, and LSAS-SR) separately (Hentati Isacsson and Johansson, 2025). These analyses primarily used Rasch analysis to reformulate each questionnaire into a set of items with adequate measurement properties (Johansson et al., 2023; Kreiner, 2007). Consequently, all questionnaires were shortened. Measurement properties were assessed in an iterative analysis process and focused on scale unidimensionality, local independence of items, ordering of response categories, and invariance, which resulted in the elimination of several items from the original scales. The reformulated MADRS-S consists of three items (originally 9), the PDSS-SR of four items (originally 7), and the LSAS-SR of eight items (originally 48). Items were primarily removed owing to issues with either multidimensionality or local dependence. Thus, 23% of all the original questions were retained (see the Supplementary material for the items retained and Hentati Isacsson and Johansson, 2025 for item details). The reformulated scale and item parameters were used to estimate the transformation of raw ordinal sum scores to interval-level latent scores. The optimization process for estimating latent score used weighted likelihood (Warm, 1989; see Hentati Isacsson and Johansson, 2025 for complete details). A Confirmatory Factor Analysis (CFA) is reported in the results to exemplify the improved psychometric properties of items in the reformulated “Rasch” versions of the original scales. The questionnaire data were obtained from the pretreatment assessment timepoint. This analysis proposed one unidimensional underlying factor implemented with lavaan (Rosseel, 2012) using oblimin rotation and the Weighted Least Squares with Mean and Variance adjustment (WLSMV) estimator. To evaluate model fit, we used scaled fit metrics and dynamic cutoffs (McNeish and Wolf, 2020a).

### Latent score

**Figure 2:**
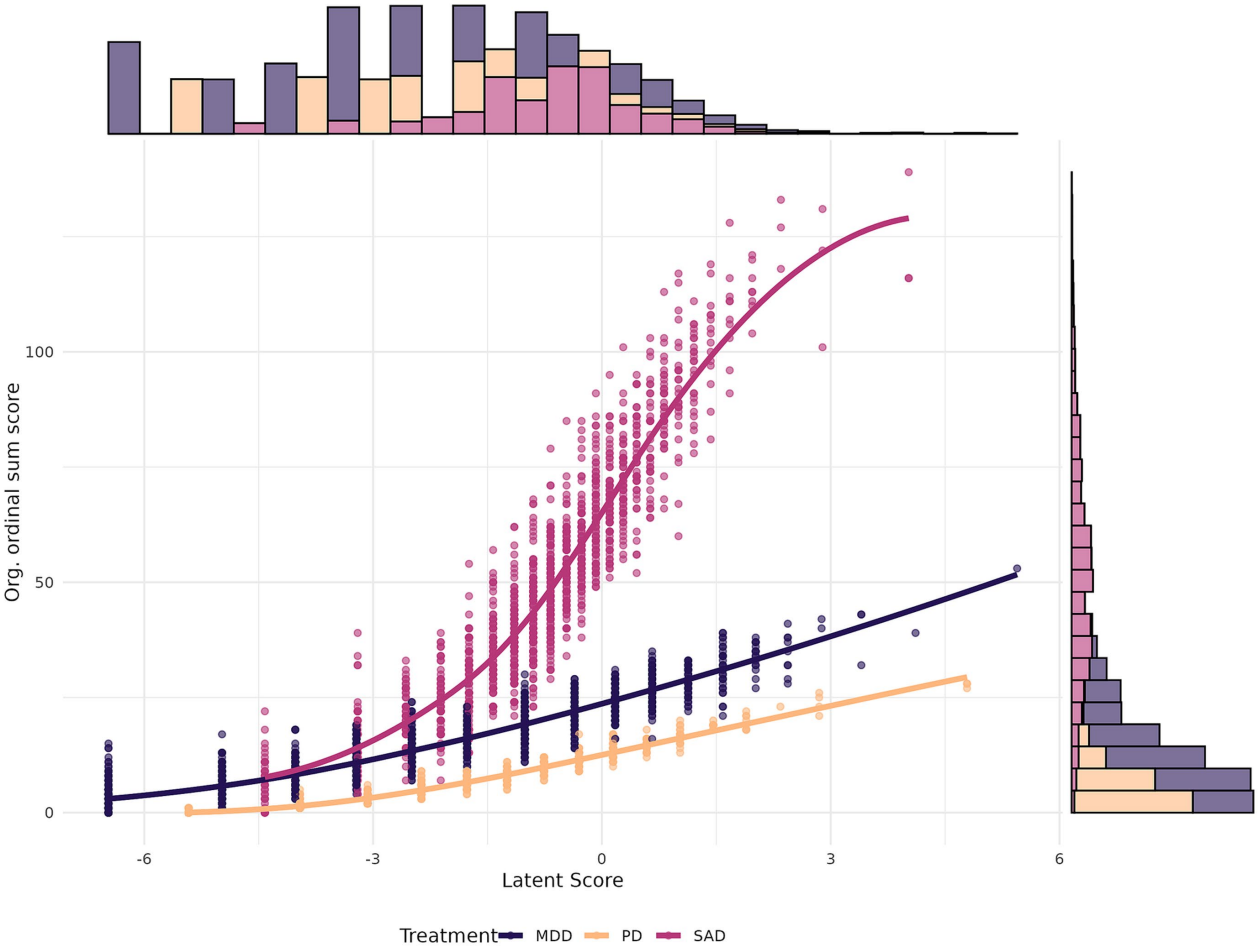
Scatterplot of sum scores and latent scores for the post-treatment self-rating. Scatterplot shows sum scores on the y-axis from the original questionnaires and latent scores of reformulated questionnaires on the x-axis. Marginal y-axis shows the histogram over the original sum scores, and the marginal x-axis shows the histogram over the latent scores from the reformulated questionnaires. A smooth curve was fitted for each treatment. Questionnaires were: Montgomery-Åsberg Depression Rating Scale Self-report for MDD, Panic Disorder Symptom Scale-Self Report for PD, and Leibowitz Social Anxiety Scale-Self report for SAD. MDD, Major Depressive Disorder, PD; Panic Disorder, SAD; Social Anxiety Disorder. This analysis was based on complete data without imputation for the post-treatment assessment, which was the predicted outcome. Pearson’s correlation was 0.92 for MDD, 0.95 for PD, and 0.90 for SAD.

The latent scores used were based on the previous RMT reformulation of the questionnaires (Hentati Isacsson and Johansson, 2025). In the predictions, the latent scores and their corresponding standard errors were used instead of the ordinal sum score from the original symptom data. As with the original symptom data, these latent scores and their standard errors were rescaled using a min-max transformation based on latent score tables, making the scale logits range from 0 to 1. Note that while the scores were rescaled, they were not standardized. See Figure 2 for a scatterplot between the original scales’ sum score and the latent scores of the Rasch reformulated scales for post-treatment time points (outcome). The data are divided based on treatments due to the clinical sample, as each questionnaire is specific to the corresponding treatment.

### Datasets

Two different datasets were created. The “Base” dataset used only the sum score of the original symptom scales for both prediction and outcome. The “Rasch” dataset used the latent score and its standard error of the reformulated symptom scale for prediction and outcome. However, the standard error of the outcome was not used, as this was tied to the outcome and would have introduced data leakage. For each dataset, the weekly symptom variables (sum or latent score) were added as separate predictors. For the Rasch datasets, the interaction between the latent score and the standard error of the latent score was also added. Furthermore, for the Rasch dataset, a weight was added only for use in the weighted regression or random forest; therefore, it was not included as a standalone predictor in the non-weighted models. This was based on the inverse sum of all the standard errors of the latent scores across the assessment times. This attempted to incorporate the estimation error for the latent variable available from the RMT analysis. Both “Base” and “Rasch” datasets used only the data from the assessment times up to and including week 4 of treatment to predict the post-treatment symptom score. As such, the pretreatment severity of symptoms was also included. The cut-off at week 4 was used, as it has previously been shown to be a good balance between clinical usefulness and predictive value (Forsell et al., 2019; Hentati Isacsson et al., 2024a). Both datasets also contained indicator variables for treatment, variables about sex and age, and the year of treatment start. These minimal clinical variables have previously been found to be useful in a predictive framework. Further statistical details can be found in the study by Hentati Isacsson et al. (2024a). Furthermore, these variables were included to counteract possible confounding in the data due to the different treatments, drift in clinical expertise over the years, sex, and age.

### Prediction models

The following models were used for analyses: A dummy regressor (DR) only predicting the mean of the outcome, Linear regression (LR), Bayesian ridge regression (BR), and a Random Forest (RF) model. No longitudinal model was used because previous findings suggest that these models do not improve predictive capability compared to their non-longitudinal counterparts (Hentati Isacsson et al., 2024b). These models were chosen based on Hentati Isacsson et al. (2024a) and Hentati Isacsson et al. (2024b), who found no differential impact of predictive models on this prediction problem, and ergo these models were all found on par with other, more computationally complex models. Furthermore, because the main objective of this study was not to determine the differential impact of predictive models on this problem, we deemed a limited number of models to suffice. All models were implemented in Python 3.10.12 (Python Software Foundation, 2023) using scikit-learn (Pedregosa et al., 2011).

The DR predicts only the mean of the outcome and represents a model that is not trained at all (a null model). LR is considered the benchmark method representing the predictive capabilities of a simple model. Bayesian Ridge Regression is a more complex model that incorporates uncertainty and regularization (Tipping, 2001). Finally, Random Forest is a machine learning model that combines multiple decision trees to improve predictive performance, reduce overfitting, and capture non-linear relationships (Breiman, 2001).

### Hyperparameters

We tuned the following hyperparameters using a grid search inside the nested cross-validation loop. BR models considered alpha_[1,2], lambda_[1,2] of [1e-6, 1e-4, 1e-2], the RF models considered all variables with 100 or 300 estimators, a minimum sample split size of [2,5,10], and a minimum sample leaf size of [1,2,5,10].

### Imputation

We imputed the missing data before cross-validation (Jaeger et al., 2020). Imputation was carried out in accordance with a multilevel imputation (Grund et al., 2018) with 20 imputations for each type of dataset using MICE implemented in R (van Buuren and Groothuis-Oudshoorn, 2011; R Core Team, 2024). Imputation allows the estimation of our models to also model the variability due to missing data, and a complete case analysis could bias our results despite our sample size (van Ginkel et al., 2020). Due to the online format of the self-rated data collection, no single items were missing, but entire questionnaires, and thus, the sum score was imputed (see Supplementary Table 1 for the number of missing data points). For the Rasch dataset, the latent score of each symptom measure was used and imputed instead of the ordinal sum of the reformulated measure. This resulted in 40 imputed datasets. The imputation was performed using a linear mixed model with predictive mean matching (2 L.pmm) (Van Buuren, 2018). To combine the results from the different imputations, Rubin’s rules were used (Van Buuren, 2018), which included the modified standard errors and degrees of freedom of the mean prediction across imputation sets to correct for the variance due to the imputation. Comparisons between models (including Welch’s *t*-test) were performed based on these means and standard errors with an alpha level of 0.05 and using two-sided tests.

### Validation

We used nested cross-validation (NCV). An NCV procedure in conjunction with multiple imputations improves the validity of confidence intervals (Bates et al., 2021). All hyperparameters were tuned in the inner CV loop to prevent overfitting (Bates et al., 2021; Cawley and Talbot, 2010). The outer CV loop consists of 10 splits, and the inner of five. Each of the 2 × 20 imputed datasets underwent the 10 × 5 CV loops. The inner CV loop determined the hyperparameter tuning, whereas the outer CV loop was used to compare the model performances.

### Prediction metrics

Primary evaluation was performed using the Root Mean Squared Error (RMSE). Based on the scaling of the symptoms 0–1 the RMSE can be interpreted as the mean percentage error in the prediction. An RMSE of 0.1 would equal, on average, 10 percentage points from the true outcome in the prediction of the continuous outcome score.

## Results

**Figure 3:**
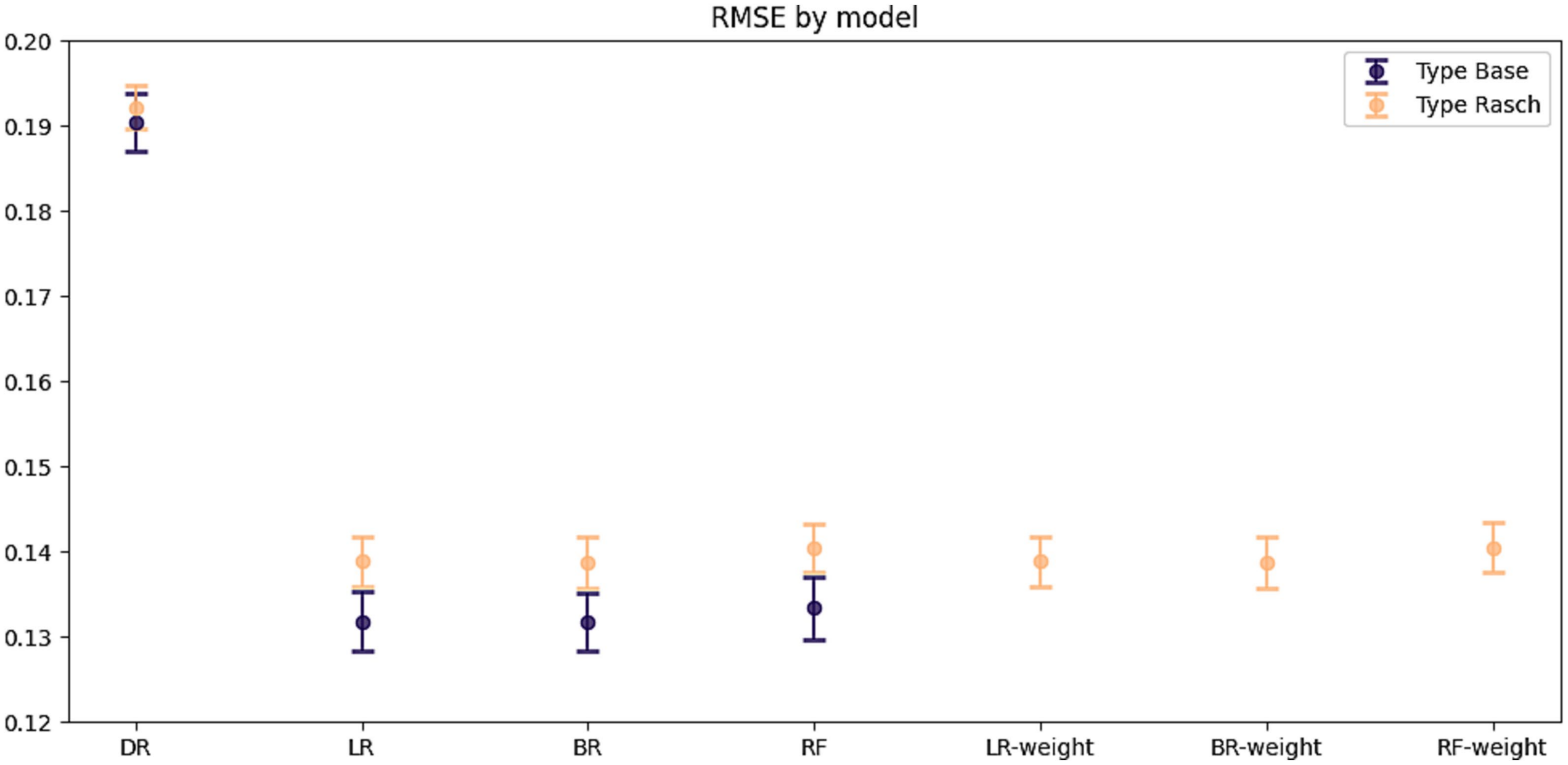
Root mean squared error for predicting symptom outcome. The root mean squared error (RMSE) mean and 95% CI were based on 20 imputed datasets for each dataset type. The RMSE can be interpreted as the mean percentage of incorrect predictions. The base dataset used the original ordinal sum scores of the questionnaires. The Rasch datasets used latent scores from psychometrically reformulated questionnaires. DR, Dummy regression, LR, Linear regression, BR, Bayesian ridge regression, RF, random forest. Weight corresponds to the weighted models using the inverse sum of the standard error for the latent scores, which was only available for the Rasch dataset.

The differences between the Base and Rasch datasets were very small, with less than a 0.0081-point difference in the RMSE score for each model in favor of the Base dataset (Figure 3). Thus, the Rasch dataset models had only a marginally worse score in RMSE, 5% (0.1389/0.1318) higher compared to the Base dataset models. For LR, the Base dataset had an RMSE of 0.1318 (95% CI, 0.1284, 0.1353), and Rasch had an RMSE of 0.1389 (95% CI, 0.1359, 0.1418) with a mean difference = −0.0070, *t*(198.57) = −3.09, *p* = 0.0023. For BR, the Base dataset had an RMSE of 0.1318 (95% CI, 0.1284, 0.1352), and Rasch had an RMSE of 0.1388 (95% CI, 0.1358, 0.1418), with a mean difference = −0.0070, *t*(205.44) = −3.04, *p* = 0.0027. For RF, Base had an RMSE of 0.1322 (95% CI, 0.1287, 0.1357), and Rasch had 0.1403 (95% CI, 0.1374, 0.1432), with a mean difference = −0.0081, *t*(218.02) = −3.51, *p* = 0.0006. All models were significantly better than the null model of the dummy regression.

Using the standard errors as weights did not improve the predictive performance of the Rasch models, having an identical performance for each of the models (Figure 3).

While the original questionnaires were less psychometrically robust (Hentati Isacsson and Johansson, 2025) than the shortened versions based on Rasch Measurement Theory, they maintained strong stochastic ordering, with higher sum scores reliably indicating (correlated) higher latent scores, as evidenced by correlations of 0.90–0.95. Table 1 shows the superior fit of the one-factor CFA for the shortened version of each questionnaire based on the RMT. We note that while some minor misspecifications also exist for the shortened version, it is always superior to the original version of the questionnaire, and the fit metrics for the shorter questionnaires had a much larger margin than the cutoffs. Thus, this CFA supports the unidimensional quality of the reformulated questionnaires but not the original versions.

**Table 1:** One-factor CFA for original items or shortened versions was based on Rasch Measurement Theory.

Questionnaire	Items	$\chi^{2}$	df	*p*	CFI	TLI	RMSEA	SRMR
MADRS-S	Org.^2^	1542.964	27	0	0.975	0.966	0.095 [0.091, 0.099]	0.043
	Rasch^*^	37.94	2	0	0.999	0.997	0.054 [0.04, 0.069]	0.009
PDSS-SR	Org.^3^	815.2	14	0	0.931	0.897	0.185 [0.174, 0.196]	0.075
	Rasch^1^	44.625	2	0	0.986	0.958	0.113 [0.085, 0.143]	0.03
LSAS-SR	Org.^3^	64324.799	1,080	0	0.533	0.513	0.191 [0.189, 0.192]	0.192
	Rasch^2^	223.817	20	0	0.96	0.944	0.08 [0.07, 0.089]	0.045

All metrics were scaled. MADRS-S was fit with one additional item to avoid a just-identified model (by adding item 6, see Hentati Isacsson and Johansson, 2025). ^*123^Indicates the level of dynamic cut-off for CFI, RMSEA, and SRMR that the model was closest to, with *indicating no model specification and 1, 2, and 3 indicating a small, medium, and large misspecification or non-conforming model to the unidimensional factor. MADRS-S, Montgomery-Åsberg Depression Rating Scale Self-report; LSAS-SR, Leibowitz Social Anxiety Scale-Self Report; PDSS-SR, Panic Disorder Symptom Scale-Self Report. Org; original items of the questionnaire. Rasch: The shortened version of the questionnaire based on Rasch analysis. 𝜒2, model chi-square; Df, model degrees of freedom; p, model *p*-value; CFI, Comparative Fit Index, higher is better; TLI, Tucker–Lewis index, higher is better; RMSEA, Root Mean Square Error of Approximation with 95% confidence intervals, lower is better; SRMR, Standardized Root Mean Square Residual, lower is better.

## Discussion

Latent scores calculated using the psychometrically optimized, substantially shorter version, comprising 23% of the full scale, showed similar predictive performance (although slightly and negligibly worse) compared to the sum score of the full scale. Overall, the original ordinal sum scores had a marginally better performance, and there were no differences in performance across models. Jacobucci and Grimm (2020) showed that varying only predictor reliability, and no other psychometric criteria, had a large impact on predictive performance, with underfitted models as a function of decreased reliability, and they did not simultaneously modify the outcome. McNamara et al. (2022), who modified both predictors and outcome at the same time, showed that there was no difference between predictive models, as was found in our results. At the same time, they showed that less reliable predictors and outcomes showed decreased predictive performance. Again, this result pertains only to modifying the noise or reliability of the predictors and outcome. Meanwhile, this study was reformulated according to four psychometric criteria using empirical data, and we do not have the same reliability estimates or control as in the simulation studies (Jacobucci and Grimm, 2020; McNamara et al., 2022).

As shown in Figure 2, the reformulated questionnaires’ latent scores have a largely simple linear relationship with the original ordinal sums. While the reformulated questionnaires are more psychometrically robust and retain essential information, the original sum scores could contain useful predictive signals despite a greater amount of noise. The results are in line with those of Sijtsma et al. (2024), which indicate similar performance between sum scores and latent scores in a predictive framework. This suggests that while the reformulated questionnaires provide a more streamlined, unidimensional measure of the trait, as shown by the CFA, some variability present in the original scores may contribute to predictive performance. This is supported by the fact that the original sum scores show stochastic ordering with largely high correlations to the (what we assume to be) underlying latent trait, as estimated using Rasch analysis. While it is not perfectly linear at the periphery of the latent continuum (Figure 2), which a non-linear model such as random forest could detect, it remains largely linear overall, reminiscent of the empirical findings of Bezruczko et al. (2016). Consequently, because the format of the outcome variable in each case aligns with the format of the predictors, the predicted relationships remain largely linear and unchanged. Thus, based on both the experimental setup in this study and previous studies, it is not entirely unexpected that the predictive performance is similar.

However, we did not interpret the higher predictive performance of the sum score as reflective of a more truthful way of handling self-rated data. As clearly argued for and shown in the study by Shmueli (2010), predictive results cannot be used to draw inferences about which models are more truthful, but rather about which could be more predictive. Another possibility is that the Rasch-improved models improved precision and reduced overfitting. This resulted in a lower predictive performance but could be more generalizable (e.g., in overpredictive or other psychiatric settings) compared to the original sum scores. This hypothesis would need to be tested with other datasets from different settings.

Furthermore, while there was a statistically significant difference in the predictive performance, the absolute difference in the RMSE score was negligible. In addition, the latent scores used only a fraction of the original questionnaire items. This was an unintended consequence of the psychometrically valid versions of the questionnaires. The latent scores’ performance was worse by 5% relative to the sum scores, corresponding to an RMSE difference of 0.70%, which is a considerable retention of information considering that only 23% of the items were retained across all questionnaires. Fewer items could be beneficial because it could significantly reduce the response burden for patients and reduce the risk of missing data, which in turn could facilitate repeated and more widespread measurements within routine care.

While we also trained models that weighted their predictions based on the inverse sum of the standard errors of the latent scores, the information from this weight did not improve the prediction compared with not using the weights. While not explicitly containing the weight variable in the non-weighted models, the non-weighted models did have access to the standard errors, which composed the weights, and these possibly had a larger influence than the composite. Future studies could explore other ways to utilize the standard errors, perhaps using another predictive framework that implements the standard error explicitly in the model (Kurz, 2023).

Since both the predictor and outcome changed simultaneously in the two conditions, a future study might investigate an independent set of predictors to predict the two different types of outcomes instead. This could possibly reveal whether a more psychometrically solid outcome variable could improve predictive performance. In addition, a future study could investigate if latent scores from the original questionnaires, without improving their psychometric qualities, have an impact on predictive performance. Furthermore, previous research has indicated improved predictive performance using items as predictors, as opposed to their summation (McNeish, 2024). While this is counter to our previous research in a similar setting (Hentati Isacsson et al., 2024a), it could be valuable in a setting that also investigates latent scores compared to a simple summation. The similar predictive performance of the latent score setup, despite using only 23% of the items, is beneficial. This study did not aim to investigate methods of shortening questionnaires and their subsequent impact on predictive performance. To investigate these aspects, another methodological setup would be needed, e.g., using the same subset of items. Such a study could also incorporate the possible impact of missing data on the analyses, such as comparisons of the impact of missing data, where one could simulate missing data and compare complete-case analyses and imputation setups. Additionally, this experimental setup could benefit from longitudinal models that take the repeated structure of the data into account, despite previous findings. Finally, there has been recent progress in predictive models with measurement errors, which indicates that it could be beneficial to predict intervals and use these instead of point predictions (Jiang and Ma, 2024).

## Conclusion

In conclusion, using empirical data from psychological treatment, our findings indicate that using latent scores as predictors and outcomes from a psychometrically improved version of the questionnaire showed similar predictive performance to the original ordinal sum scores. While the psychometric properties were improved by the Rasch analyses, it is inconclusive whether this also improved precision and reduced overfitting or if the Base dataset retained useful variability. For the models using the latent score, their predictive performance was marginally reduced by 5% (a 0.70% RMSE increase) while using only 23% of the original items. This suggests that while reformulated questionnaires can streamline measurement and lower the burden on patients, their impact on improving predictive performance in this study was limited.

## Data Availability

The original contributions presented in the study are included in the article/Supplementary material, further inquiries can be directed to the corresponding author.

## References

[ref1] Andrich D. Marais I. (2019). A course in Rasch measurement theory: measuring in the educational, social and health sciences. Singapore: Springer Singapore.

[ref2] Barkham M. De Jong K. Delgadillo J. Lutz W. (2023). Routine outcome monitoring (ROM) and feedback: research review and recommendations. Psychother. Res. 33, 841–855. doi: 10.1080/10503307.2023.2181114, 36931228

[ref3] Bates S. Hastie T. Tibshirani R. (2021). Cross-validation: what does it estimate and how well does it do it?. arXiv:2104.00673. 10.1080/01621459.2023.2197686 PMC11412612 39308484

[ref4] Bezruczko N. Fatani S. S. Magari N. (2016). Three Tales of change: ordinal scores, Residualized gains, and Rasch logits—when are they interchangeable? SAGE Open 6:2158244016659905. doi: 10.1177/2158244016659905

[ref5] Breiman L. (2001). Random forests. Mach. Learn. 45, 5–32.

[ref6] Cawley G. C. Talbot N. L. C. (2010). On over-fitting in model selection and subsequent selection Bias in performance evaluation. J. Mach. Learn. Res. 11, 2079–2107. doi: 10.5555/1756006.185992110.5555

[ref7] Christensen K. B. Kreiner S. Mesbah M. (2013). Rasch models in health. Great Britain, United States: ISTE Ltd and John Wiley & Sons.

[ref8] Cohen P. Cohen J. Aiken L. S. West S. G. (1999). The problem of units and the circumstance for POMP. Multivar. Behav. Res. 34, 315–346. doi: 10.1207/S15327906MBR3403_2

[ref9] Collins G. S. Moons K. G. M. Dhiman P. Riley R. D. Beam A. L. Calster B. V. . (2024). TRIPOD+AI statement: updated guidance for reporting clinical prediction models that use regression or machine learning methods. BMJ 385:e078378. doi: 10.1136/bmj-2023-078378, 38626948 PMC11019967

[ref10] El Alaoui S. Hedman E. Kaldo V. Hesser H. Kraepelien M. Andersson E. . (2015). Effectiveness of internet-based cognitive–behavior therapy for social anxiety disorder in clinical psychiatry. J. Consult. Clin. Psychol. 83, 902–914. doi: 10.1037/a0039198 26009780

[ref11] Elson M. Hussey I. Alsalti T. Arslan R. C. (2023). Psychological measures aren’t toothbrushes. Commun. Psychol. 1, 1–4. doi: 10.1038/s44271-023-00026-9, 39242966 PMC11332227

[ref12] Fisher W. P. Burton E. C. (2010). Embedding measurement within existing computerized data systems: scaling clinical laboratory and medical records heart failure data to predict ICU admission. J. Appl. Meas. 11, 271–287. 20847475

[ref13] Flake J. K. Davidson I. J. Wong O. Pek J. (2022). Construct validity and the validity of replication studies: a systematic review. Am. Psychol. 77, 576–588. doi: 10.1037/amp0001006, 35482669

[ref14] Flake J. K. Fried E. I. (2020). Measurement schmeasurement: questionable measurement practices and how to avoid them. Adv. Methods Pract. Psychol. Sci. 3, 456–465. doi: 10.1177/2515245920952393

[ref15] Forsell E. Jernelöv S. Blom K. Kraepelien M. Svanborg C. Andersson G. . (2019). Proof of concept for an adaptive treatment strategy to prevent failures in internet-delivered CBT: a single-blind randomized clinical trial with insomnia patients. Am. J. Psychiatry 176, 315–323. doi: 10.1176/appi.ajp.2018.18060699, 30696270

[ref16] Fresco D. M. Coles M. E. Heimberg R. G. Liebowitz M. R. Hami S. Stein M. B. . (2001). The Liebowitz social anxiety scale: a comparison of the psychometric properties of self-report and clinician-administered formats. Psychol. Med. 31, 1025–1035. doi: 10.1017/S0033291701004056, 11513370

[ref17] Grund S. Lüdtke O. Robitzsch A. (2018). Multiple imputation of missing data for multilevel models: simulations and recommendations. Organ. Res. Methods 21, 111–149. doi: 10.1177/1094428117703686

[ref18] Hedman E. Ljótsson B. Kaldo V. Hesser H. El Alaoui S. Kraepelien M. . (2014). Effectiveness of internet-based cognitive behaviour therapy for depression in routine psychiatric care. J. Affect. Disord. 155, 49–58. doi: 10.1016/j.jad.2013.10.023, 24238951

[ref19] Hedman E. Ljótsson B. Rück C. Bergström J. Andersson G. Kaldo V. . (2013). Effectiveness of internet-based cognitive behaviour therapy for panic disorder in routine psychiatric care. Acta Psychiatr. Scand. 128, 457–467. doi: 10.1111/acps.12079, 23406572

[ref20] Hentati Isacsson N. Ben Abdesslem F. Forsell E. Boman M. Kaldo V. (2024a). Methodological choices and clinical usefulness for machine learning predictions of outcome in internet-based cognitive behavioural therapy. Commun. Med. 4, 1–11. doi: 10.1038/s43856-024-00626-4, 39384934 PMC11464669

[ref21] Hentati Isacsson N. Johansson M. (2025). Three psychometric evals. Available online at: https://intraverbal.github.io/

[ref22] Hentati Isacsson N. Zantvoort K. Forsell E. Boman M. Kaldo V. (2024b). Making the most out of timeseries symptom data: a machine learning study on symptom predictions of internet-based CBT. Internet Interv. 38:100773. doi: 10.1016/j.invent.2024.100773, 39310714 PMC11416613

[ref23] Houck P. R. Spiegel D. A. Shear M. K. Rucci P. (2002). Reliability of the self-report version of the panic disorder severity scale. Depress. Anxiety 15, 183–185. doi: 10.1002/da.10049, 12112724

[ref24] Jacobucci R. Grimm K. J. (2020). Machine learning and psychological research: the unexplored effect of measurement. Perspect. Psychol. Sci. 15, 809–816. doi: 10.1177/1745691620902467, 32348703

[ref25] Jaeger B. C. Tierney N. J. Simon N. R. (2020). When to impute? Imputation before and during cross-validation. arXiv:2010.00718 [Cs, Stat].

[ref26] Jiang F. Ma Y. (2024). Prediction in measurement error models (no. arXiv:2405.10461; version 1) arXiv. doi: 10.48550/arXiv.2405.10461

[ref27] Johansson M. Preuter M. Karlsson S. Möllerberg M.-L. Svensson H. Melin J. (2023). Valid and reliable? Basic and expanded recommendations for psychometric reporting and quality assessment: OSF.

[ref28] Kreiner S. (2007). Validity and objectivity: reflections on the role and nature of Rasch models. Nord. Psychol. 59, 268–298. doi: 10.1027/1901-2276.59.3.268

[ref29] Kurz A. S. (2023). Statistical rethinking with brms, ggplot2, and the tidyverse: Second edition (version 0.4.0). Available online at: https://bookdown.org/content/4857/

[ref30] Lilienfeld S. O. Strother A. N. (2020). Psychological measurement and the replication crisis: four sacred cows. Can. Psychol. Psychol. Can. 61, 281–288. doi: 10.1037/cap0000236

[ref31] Luijken K. Groenwold R. H. H. Van Calster B. Steyerberg E. W. van Smeden M. (2019). Impact of predictor measurement heterogeneity across settings on the performance of prediction models: a measurement error perspective. Stat. Med. 38, 3444–3459. doi: 10.1002/sim.8183, 31148207 PMC6619392

[ref32] McNamara M. E. Zisser M. Beevers C. G. Shumake J. (2022). Not just “big” data: importance of sample size, measurement error, and uninformative predictors for developing prognostic models for digital interventions. Behav. Res. Ther. 153:104086. doi: 10.1016/j.brat.2022.104086, 35462242

[ref33] McNeish D. (2018). Thanks coefficient alpha, we’ll take it from here. Psychol. Methods 23, 412–433. doi: 10.1037/met0000144, 28557467

[ref34] McNeish D. (2022). Limitations of the sum-and-alpha approach to measurement in behavioral research. Policy Insights Behav. Brain Sci. 9, 196–203. doi: 10.1177/23727322221117144

[ref35] McNeish D. (2024). Practical implications of sum scores being psychometrics’ greatest accomplishment. Psychometrika 89, 1148–1169. doi: 10.1007/s11336-024-09988-z, 39031300

[ref36] McNeish D. Wolf M. G. (2020a). Dynamic fit index cutoffs for confirmatory factor analysis models: OSF. 10.1037/met0000425 34694832

[ref37] McNeish D. Wolf M. G. (2020b). Thinking twice about sum scores. Behav. Res. Methods 52, 2287–2305. doi: 10.3758/s13428-020-01398-0, 32323277

[ref38] Michell J. (1997). Quantitative science and the definition of measurement in psychology. Br. J. Psychol. 88, 355–383. doi: 10.1111/j.2044-8295.1997.tb02641.x

[ref39] Montgomery S. A. Asberg M. (1979). A new depression scale designed to be sensitive to change. Br. J. Psychiatry 134, 382–389. doi: 10.1192/bjp.134.4.382, 444788

[ref40] Pedregosa F. Varoquaux G. Gramfort A. Michel V. Thirion B. Grisel O. . (2011). Scikit-learn: machine learning in Python. J. Mach. Learn. Res. 12, 2825–2830. doi: 10.1023/A:1010933404324

[ref41] Pendrill L. R. (2018). Assuring measurement quality in person-centred healthcare. Meas. Sci. Technol. 29:034003. doi: 10.1088/1361-6501/aa9cd2

[ref42] Pendrill L. R. Melin J. Stavelin A. Nordin G. (2023). Modernising receiver operating characteristic (ROC) curves. Algorithms 16:1–22. doi: 10.3390/a16050253

[ref43] Python Software Foundation (2023) Python programming language. Available online at: https://www.python.org/

[ref44] R Core Team (2024) R: a language and environment for statistical computing R foundation for statistical computing. Available online at: https://www.R-project.org/ (Accessed December, 12, 2025).

[ref45] Rosseel Y. (2012). Lavaan: an R package for structural equation modeling. J. Stat. Softw. 48, 1–36. doi: 10.18637/jss.v048.i02

[ref46] Shmueli G. (2010). To explain or to predict? Stat. Sci. 25, 289–310. doi: 10.1214/10-STS330

[ref47] Sijtsma K. Ellis J. L. Borsboom D. (2024). Recognize the value of the sum score, psychometrics’ greatest accomplishment. Psychometrika 89, 84–117. doi: 10.1007/s11336-024-09964-7, 38627311 PMC11588849

[ref48] Tipping M. E. (2001). Sparse Bayesian learning and the relevance vector machine. J. Mach. Learn. Res. 1, 211–244.

[ref49] Titov N. Dear B. Nielssen O. Staples L. Hadjistavropoulos H. Nugent M. . (2018). ICBT in routine care: a descriptive analysis of successful clinics in five countries. Internet Interv. 13, 108–115. doi: 10.1016/j.invent.2018.07.006, 30206525 PMC6112100

[ref50] Van Buuren S. (2018). Flexible imputation of missing data. 2nd Edn: CRC press. https://stefvanbuuren.name

[ref51] van Buuren S. Groothuis-Oudshoorn K. (2011). Mice: multivariate imputation by chained equations in R. J. Stat. Softw. 45, 1–67. doi: 10.18637/jss.v045.i03

[ref52] van Ginkel J. R. Linting M. Rippe R. C. A. van der Voort A. (2020). Rebutting existing misconceptions about multiple imputation as a method for handling missing data. J. Pers. Assess. 102, 297–308. doi: 10.1080/00223891.2018.1530680, 30657714

[ref53] Warm T. A. (1989). Weighted likelihood estimation of ability in item response theory. Psychometrika 54, 427–450. doi: 10.1007/BF02294627

[ref54] Widaman K. F. Revelle W. (2023). Thinking thrice about sum scores, and then some more about measurement and analysis. Behav. Res. Methods 55, 788–806. doi: 10.3758/s13428-022-01849-w, 35469086 PMC10027776

[ref55] Zantvoort K. Hentati Isacsson N. Funk B. Kaldo V. (2024). Dataset size versus homogeneity: a machine learning study on pooling intervention data in e-mental health dropout predictions. DIGITAL HEALTH 10:20552076241248920. doi: 10.1177/20552076241248920, 38757087 PMC11097733

