# Supplementary material for: Latent trait or sum score: addressing measurement challenges in the prediction of self-rated symptom outcomes in psychological treatment
Source: Front Psychol. 2026 Feb 26;17:1654996. doi: 10.3389/fpsyg.2026.1654996 (PMC12979473; doi:10.3389/fpsyg.2026.1654996)
Supplement: Supplementary file 1 [file Table_1.DOCX]

Supplement document:

**Manuscript**

Latent Trait or Sum Score: Addressing Measurement Challenges in the Prediction of Self-Rated Symptom Outcomes in Psychological Treatment**Note**

This supplement contains more details for the main paper, including link to raw data.

The entire code for prediction, model tuning and result processing can be found at: https://osf.io/3hcw8/?view_only=4817ab46a6c94b4bb17281c4c4398d8d

**Contents**

**Items retained**

**Imputation diagnostics and number of imputed values**

Table 1

**Supplementary files**

Following files found at: https://osf.io/3hcw8/?view_only=4817ab46a6c94b4bb17281c4c4398d8d

1. Code (rar file), includes results for all metrics and item parameters.

2. Results all metrics (csv file)

**Items retained**

Montgomery-Åsberg Depression Rating Scale-Self report (MADRS-S): Items 1, 7, & 9.

Panic Disorder Symptom Scale-Self Report (PDSS-SR): Items 1, 3, 4, & 6.

Leibowitz Social Anxiety Scale-Self Report version (LSAS-SR): Items 2b, 5b, 8a, 10a, 14a, 18b, 19a, & 24a.

**Imputation diagnostics and number of imputed values**

| Table 1. Imputation diagnostics and number of imputed values | | | | |
| --- | --- | --- | --- | --- |
| Variable | n fraction | n | influx | outflux |
| MADRS-S screening | 1.00 | 6461 | 0.00 | 1.00 |
| LSAS-SR screening | 0.92 | 5970 | 0.06 | 0.85 |
| PDSS-SR screening | 0.96 | 6200 | 0.03 | 0.91 |
| Mainsymptom Pre-treatment | 0.96 | 6217 | 0.02 | 0.91 |
| Mainsymptom Week 1 | 0.82 | 5319 | 0.12 | 0.58 |
| Mainsymptom Week 2 | 0.85 | 5467 | 0.09 | 0.59 |
| Mainsymptom Week 3 | 0.83 | 5355 | 0.10 | 0.54 |
| Mainsymptom Week 4 | 0.81 | 5232 | 0.11 | 0.50 |
| Mainsymptom Week 5 | 0.77 | 4983 | 0.14 | 0.42 |
| Mainsymptom Week 6 | 0.74 | 4788 | 0.17 | 0.37 |
| Mainsymptom Week 7 | 0.71 | 4558 | 0.20 | 0.31 |
| Mainsymptom Week 8 | 0.68 | 4366 | 0.22 | 0.27 |
| Mainsymptom Week 9 | 0.64 | 4149 | 0.26 | 0.23 |
| Mainsymptom Week 10 | 0.60 | 3909 | 0.30 | 0.20 |
| Mainsymptom Post-treatment* | 0.76 | 4925 | 0.16 | 0.45 |
| *Post-treatment is the outcome.  Total number of included participants was 6464. Mainsymptom Week 4 – 10 was not used in the predictive model, only for imputation.  n is the number of observed datapoints for that variable. n fraction is the proportion of observed compared to the total number of included participants. MADRS-S; Montgomery-Åsberg Depression Rating Scale Self-report, LSAS-SR; Leibowitz Social Anxiety Scale-Self report, PDSS-SR; Panic Disorder Symptom Scale-Self Report. Influx; Influx depends on the amount of missing, it is 0 for complete data, if the amount of missing is equal the variable with higher influx is better connected to observed data. Outflux; Outflux depends on also on the amount of missing, it is 1 for complete data, if the amount of missing is equal the variable with a higher outflux is better connected to missing data and more useful for imputing. All weeks were included in the imputation procedure to increase the precision of the imputed values. | | | | |
