# Supplementary figures and images for: Latent trait or sum score: addressing measurement challenges in the prediction of self-rated symptom outcomes in psychological treatment
Source: Front Psychol. 2026 Feb 26;17:1654996. doi: 10.3389/fpsyg.2026.1654996 (PMC12979473; doi:10.3389/fpsyg.2026.1654996)

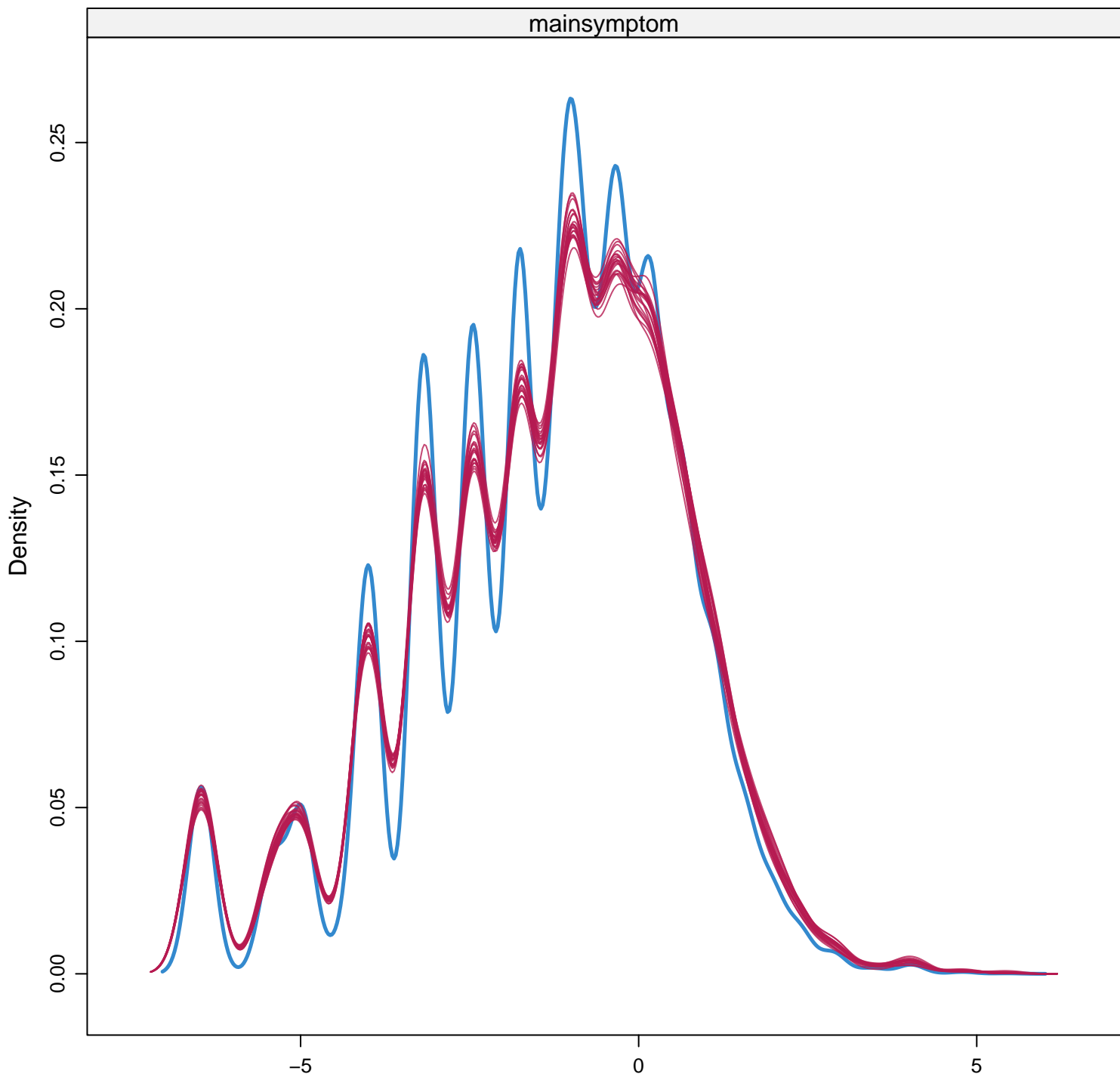

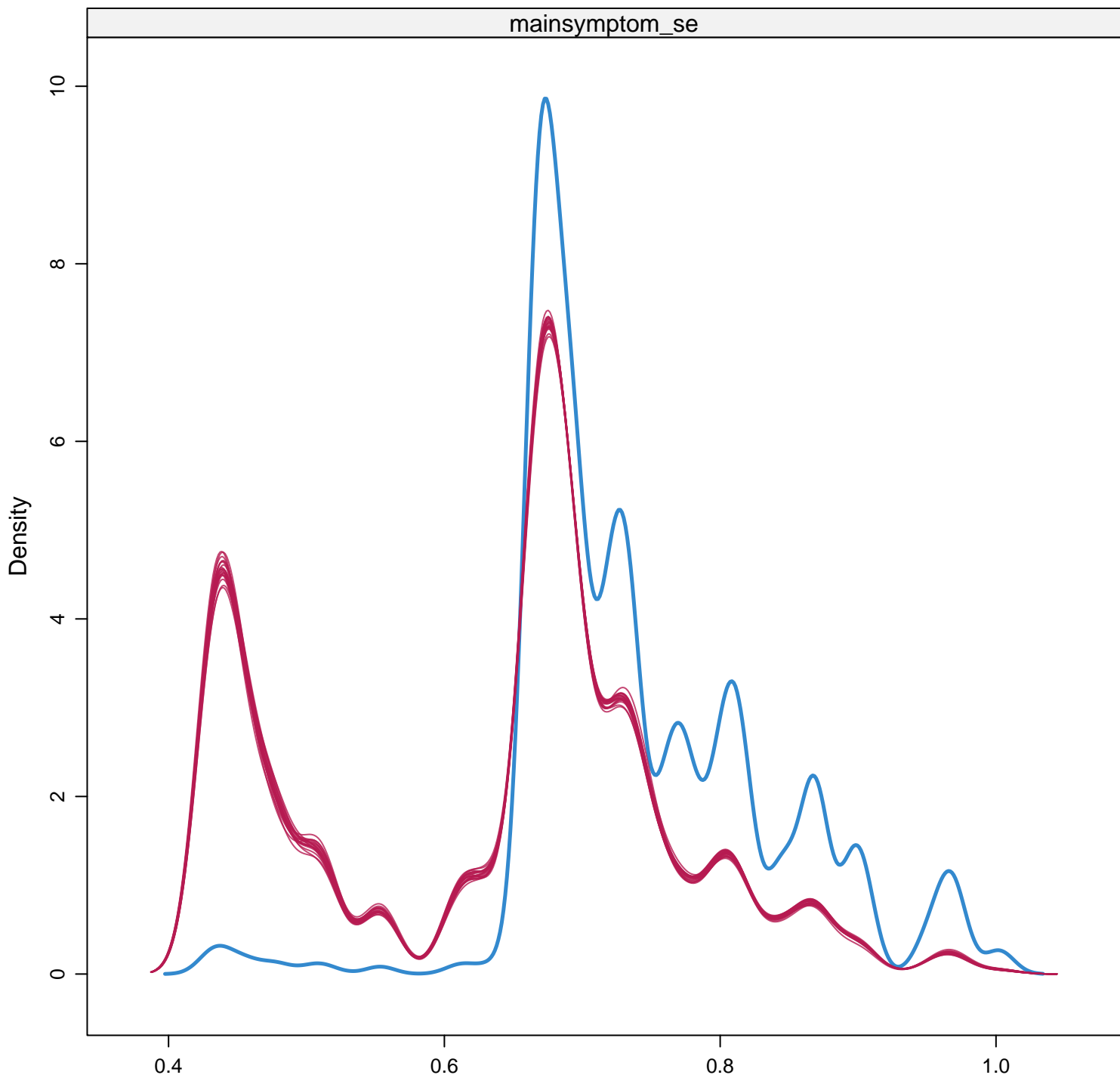

MADRS.S\_sum\_SCREEN

Density

0.25  
0.20  
0.15  
0.10  
0.05  
0.00

-10

-5

0

5

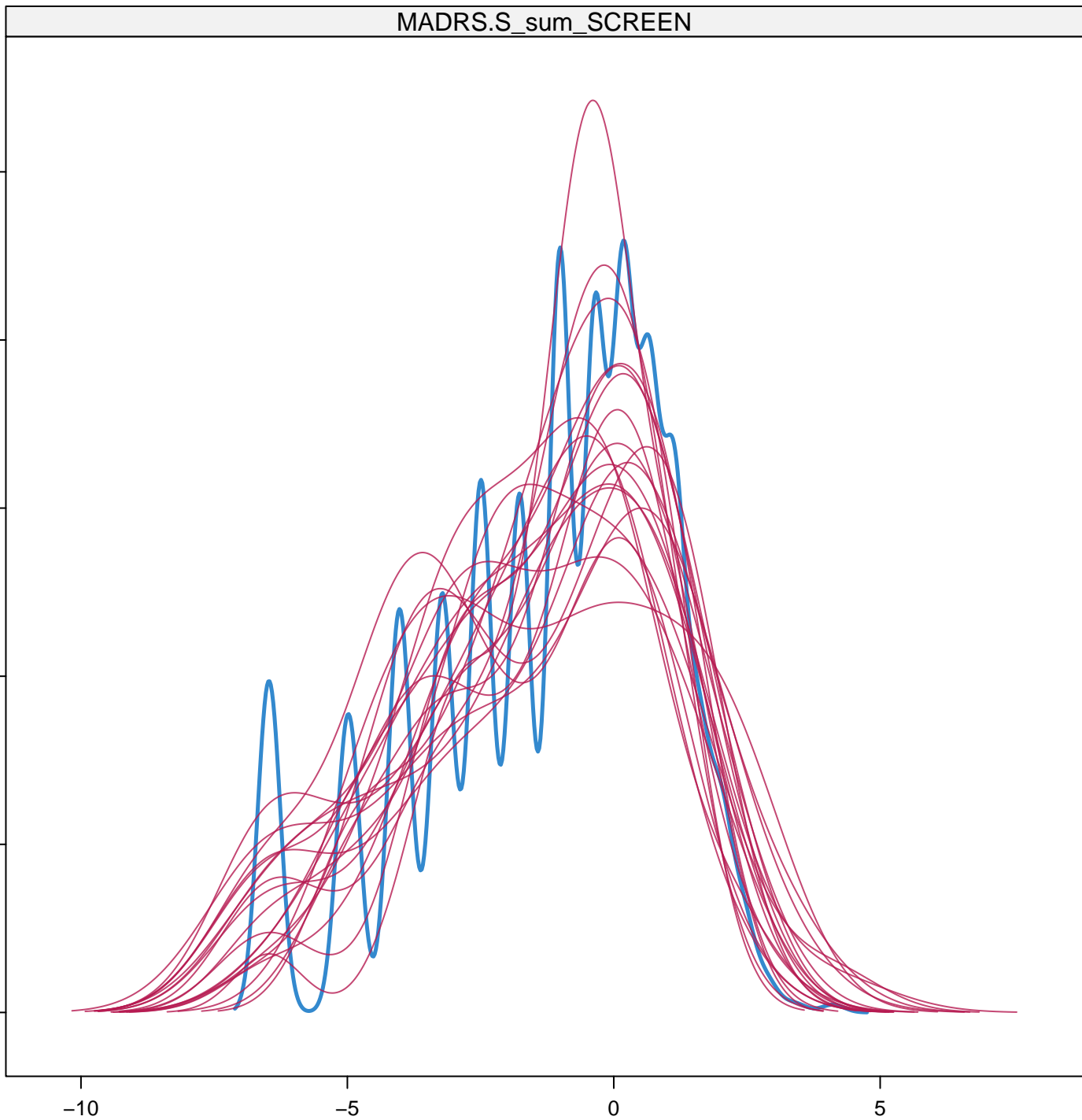

Density

0.3

0.2

0.1

0.0

-5

0

5

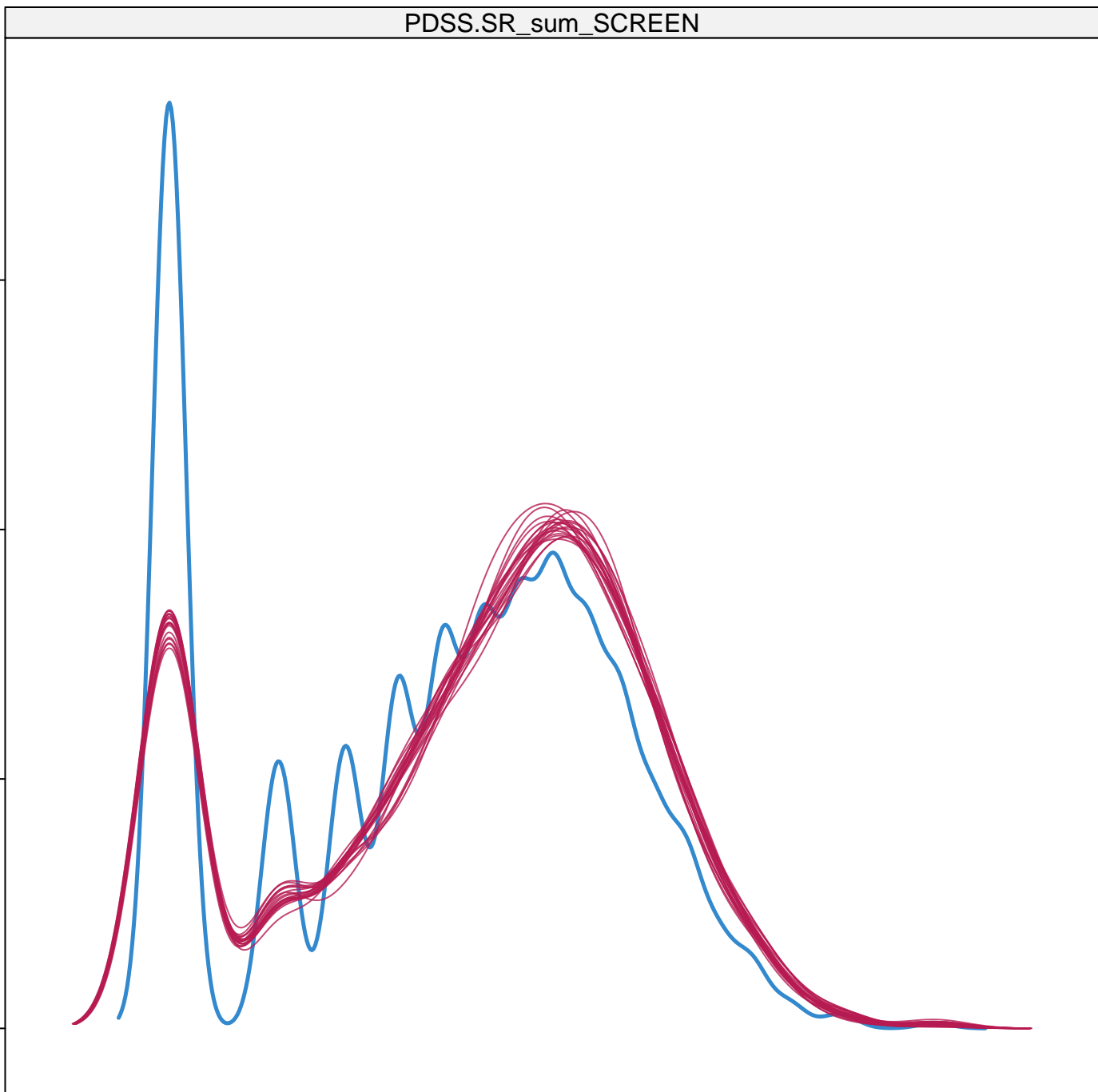

LSAS.SR\_sum\_SCREEN

Density

0.3  
0.2  
0.1  
0.0

-4

-2

0

2

4

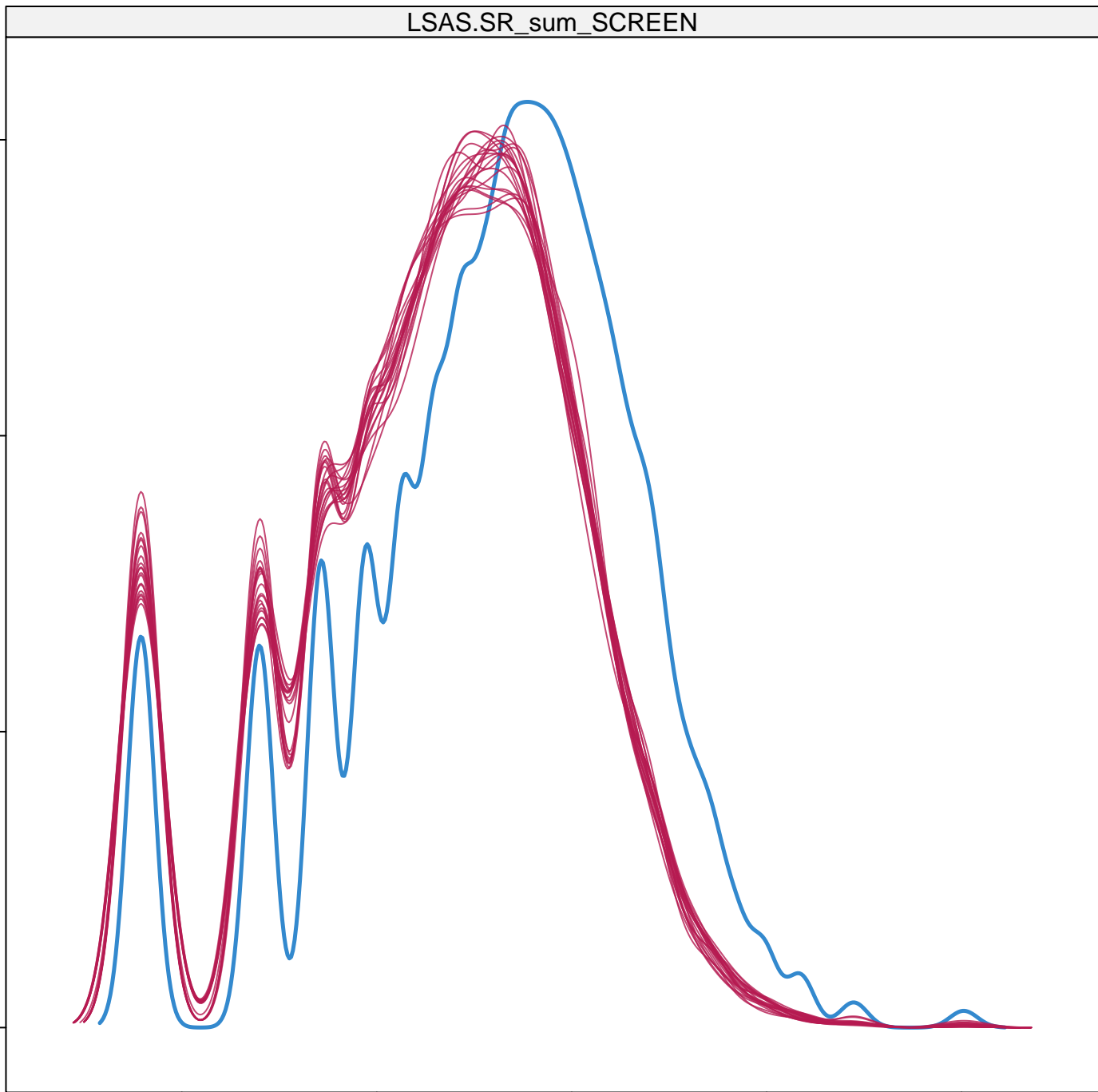

Supplement: Supplementary file 3 [file Data_Sheet_2.ZIP › rasch_prediction/imputation/results/rasch_density_imputation.pdf]

mainsymptom

Density

0.03  
0.02  
0.01  
0.00

0

50

100

150

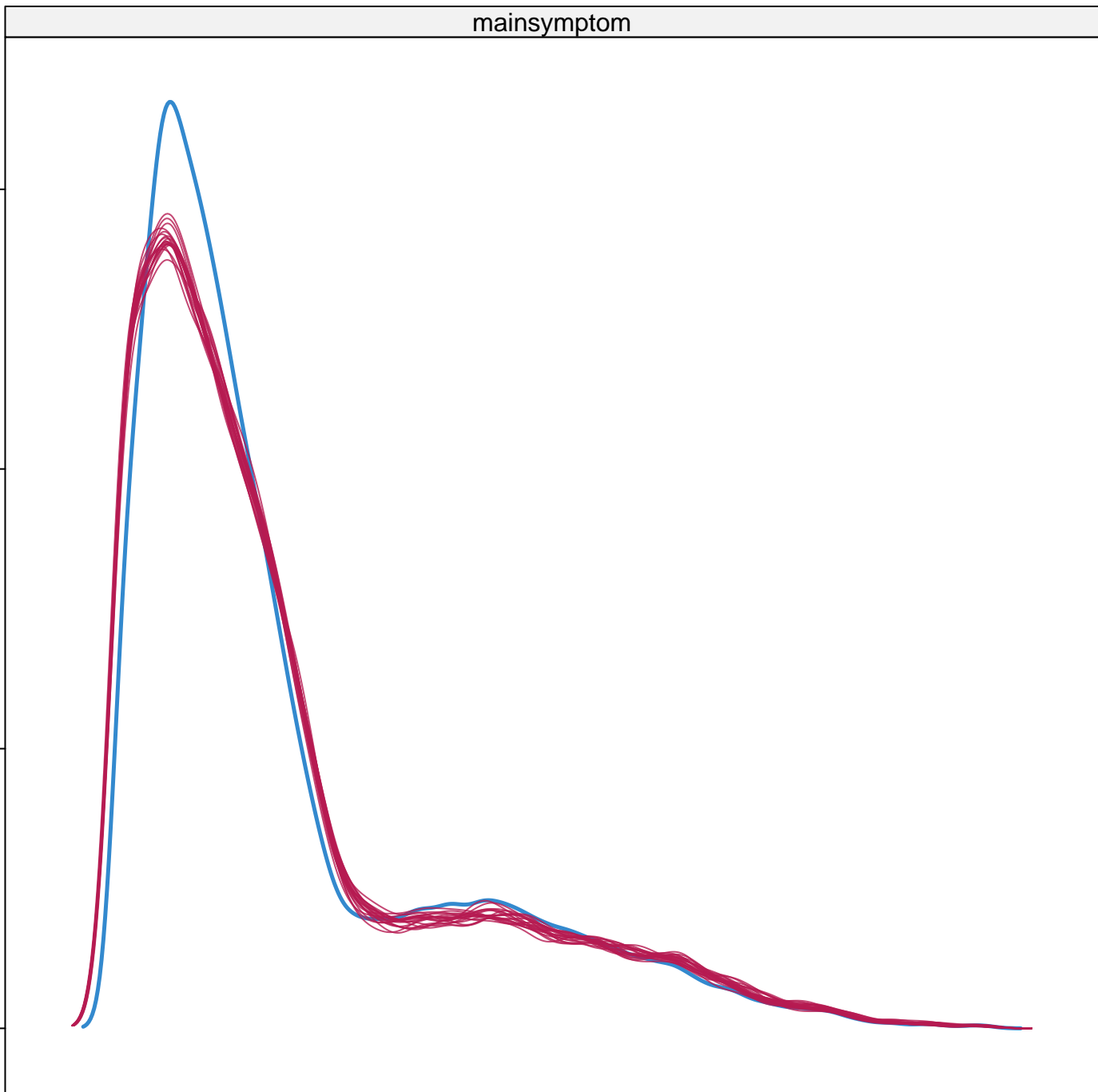

MADRS.S\_sum\_SCREEN

Density

0.06  
0.04  
0.02  
0.00

-10 0 10 20 30 40 50

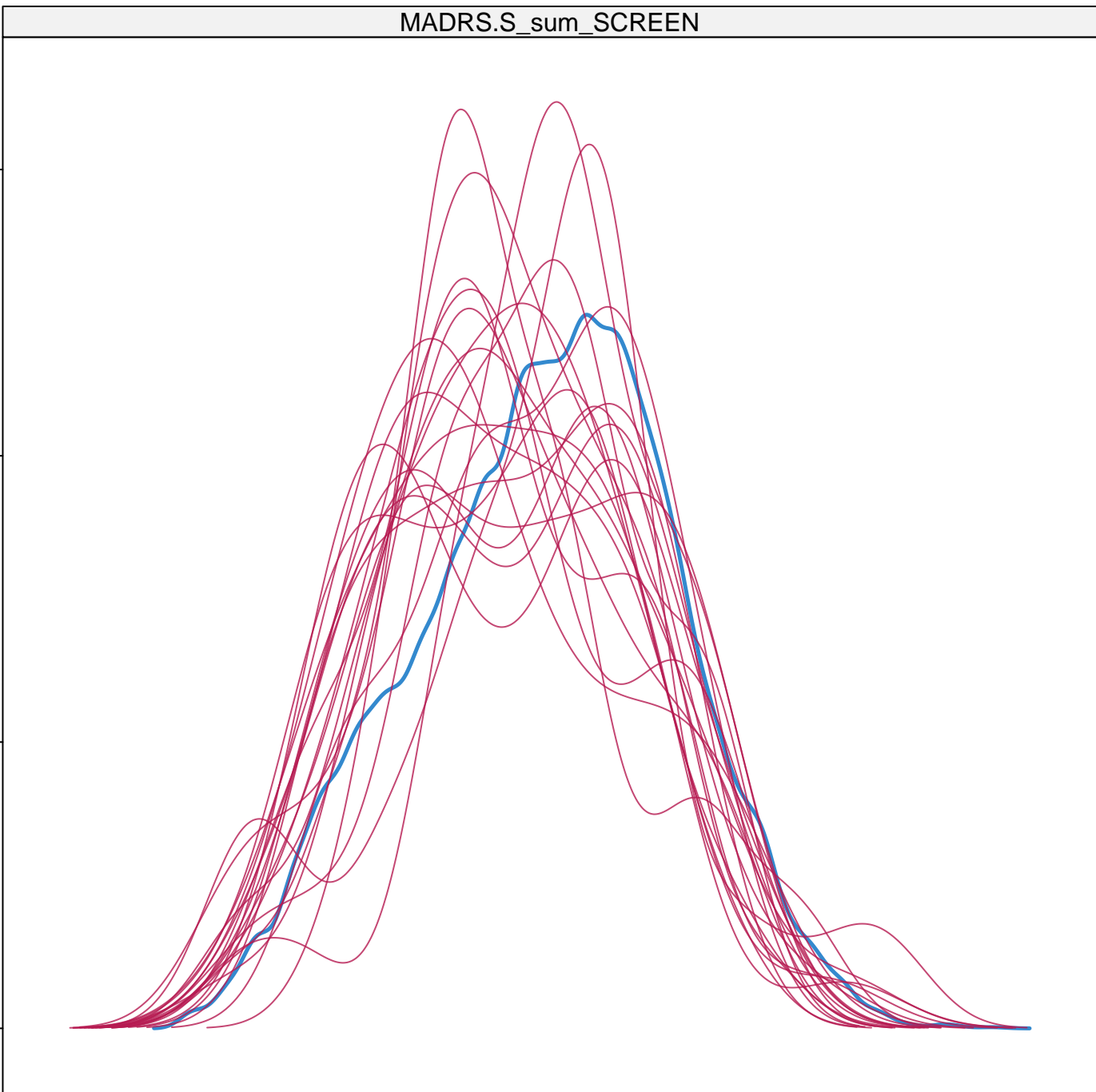

Density

0.10

0.05

0.00

0

10

20

30

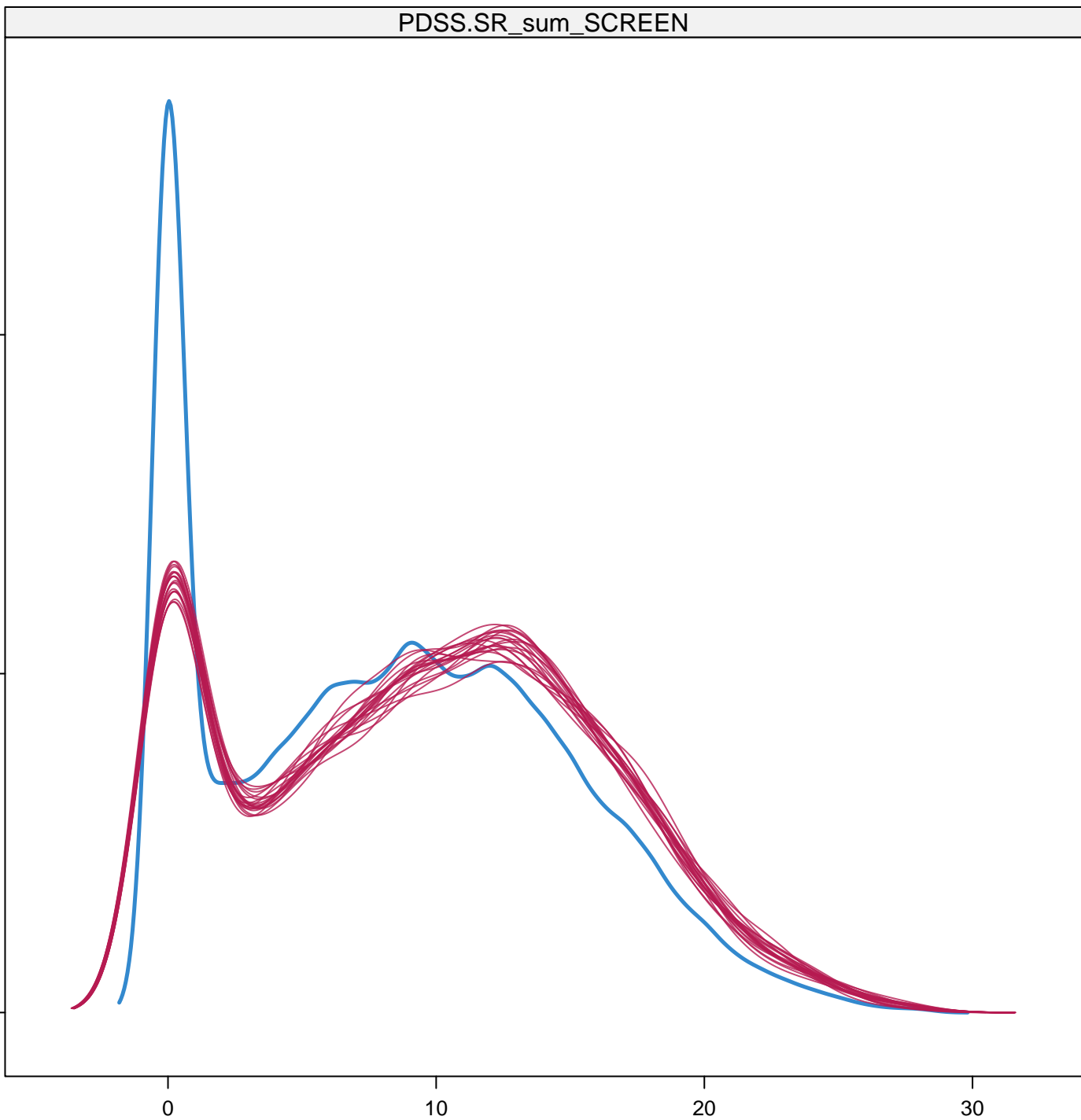

LSAS.SR\_sum\_SCREEN

Density

0.015

0.010

0.005

0.000

0

50

100

150

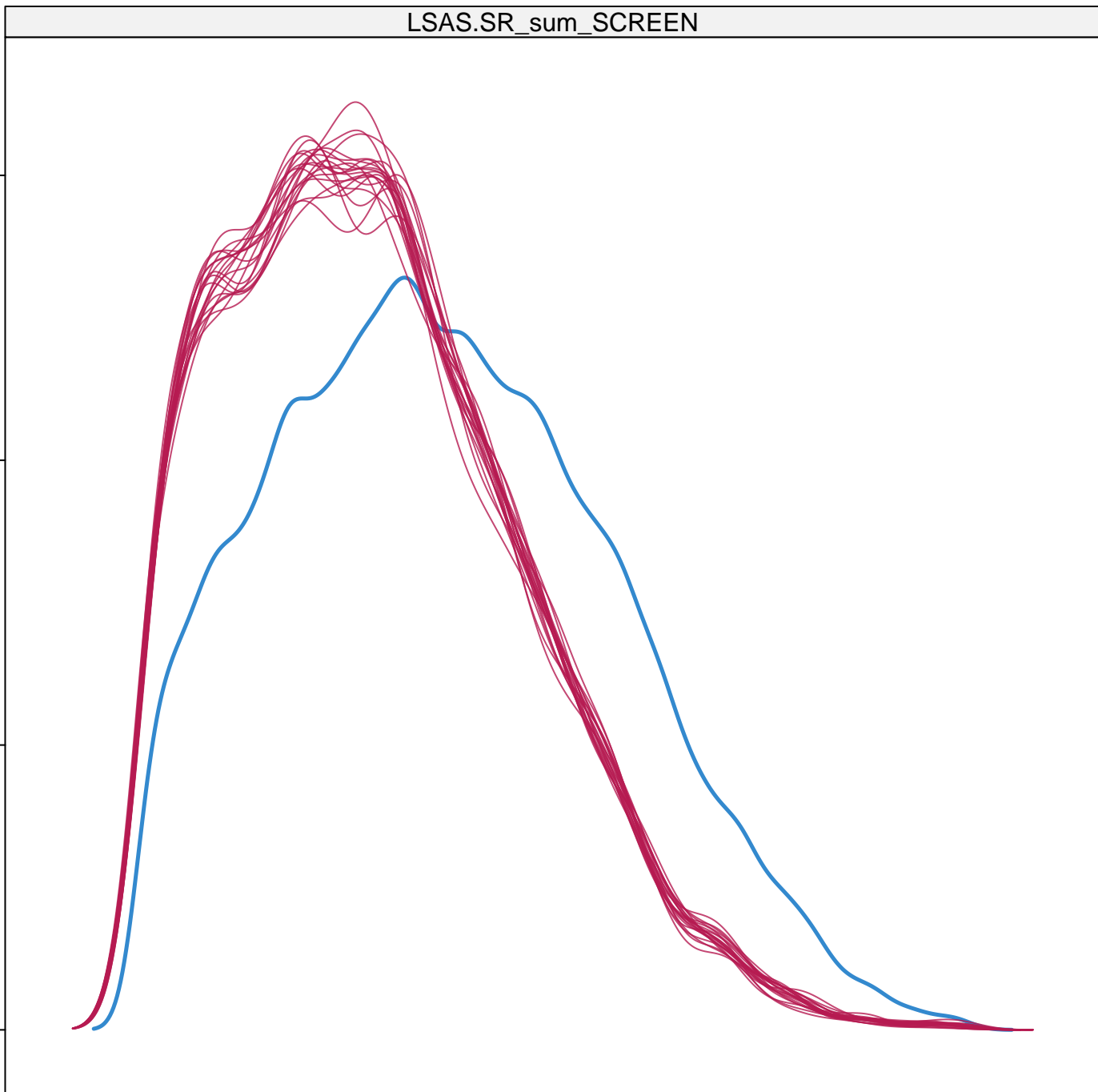

Supplement: Supplementary file 3 [file Data_Sheet_2.ZIP › rasch_prediction/imputation/results/base_density_imputation.pdf]
